# peakScout—a biologist-friendly tool for bidirectional peak-gene mapping

**DOI:** 10.1093/bioinformatics/btag653

**Published:** 2026-09-07

**Authors:** Alexander L Lin, Lana A Cartailler, Jean-Philippe Cartailler

**Affiliations:** Creative Data Solutions, Center for Stem Cell Biology, School of Medicine Basic Sciences, Vanderbilt University, Nashville, TN 37027, United States; Creative Data Solutions, Center for Stem Cell Biology, School of Medicine Basic Sciences, Vanderbilt University, Nashville, TN 37027, United States; Creative Data Solutions, Center for Stem Cell Biology, School of Medicine Basic Sciences, Vanderbilt University, Nashville, TN 37027, United States

## Abstract

**Motivation:**

Translating genomic peak data into biologically meaningful knowledge typically requires bioinformatics expertise, creating a barrier for non-technical users. We developed peakScout to bridge the gap between peaks, genes, and gene annotations, enabling users to quickly focus on biological context rather than bioinformatics skills, in a reproducible and robust fashion.

**Results:**

peakScout is a command line and web-based program that performs bidirectional mapping between genomic peaks and genes. The peak-to-gene mode identifies which genes are potentially regulated by specific genomic regions, while the gene-to-peak mode reveals which regulatory elements might influence particular genes of interest. An algorithm for nearest-feature detection handles the complex spatial relationships between genomic elements, considering factors like distance constraints and feature overlaps.

**Availability and implementation:**

The web version of peakScout is available at https://vandydata.github.io/peakScout/. The command line version is available at https://github.com/vandydata/peakScout and archived on Zenodo (https://doi.org/10.5281/zenodo.21211605) under the GNU Affero General Public License v3.0. Installation instructions, example datasets, and usage examples are provided in the GitHub repository README file.

## 1 Introduction

High-throughput genomic techniques have revolutionized our understanding of protein-DNA interactions, chromatin modifications, and gene regulation. Methods such as ChIP-seq, CUT&RUN, CUT&TAG, ATAC-seq, DNase-seq, and FAIRE-seq typically generate thousands of genomic regions of interest (peaks) that represent binding sites, accessible chromatin regions, or enriched chromatin marks. However, translating these genomic coordinates into biologically meaningful insights remains challenging for researchers without bioinformatics expertise.

While several tools exist for peak-gene association, they differ in supported query directions and accessibility. BEDTools (Quinlan and Hall 2010) provides flexible interval operations via the command line and can approximate peak-to-gene mapping using closestBed, but gene-to-peak queries require custom scripting and format conversion. GREAT (McLean *et al.* 2010) performs region-based functional enrichment but does not support gene-to-peak retrieval. ChIPseeker (Yu *et al.* 2015) provides peak annotation with k-nearest gene reporting but is peak-centric and requires R and Bioconductor. HOMER (Heinz *et al.* 2010) similarly annotates peaks to genes but does not expose a gene-to-peak query. peakScout addresses this gap by providing both peak-to-gene and gene-to-peak as native, symmetric operations, accessible through either web or command line interfaces, with direct support for MACS2 (Zhang *et al*. 2008), SEACR (Meers *et al*. 2019), and BED6 formats (Table S1). The tool supports both peak-to-gene and gene-to-peak analyses, allowing researchers to either identify potential target genes of regulatory elements or find regulatory elements that might influence specific genes of interest, with output available in flexible CSV and Excel files.

By simplifying this critical analytical step, peakScout enables bench scientists and bioinformatics novices to quickly interpret their genomic data without extensive computational training. Its accessible output formats facilitate rapid integration with downstream analyses and visualization tools, accelerating the path from raw peak data to biological insights.

## 2 Materials and methods

peakScout is implemented in Python, leveraging the high-performance Polars data processing library for efficient manipulation of tabular genomic data. The core algorithmic approach in peakScout centers around efficient chromosome-specific feature decomposition and nearest-feature detection. For reference data management, peakScout employs a hierarchical decomposition strategy that organizes genomic features by type (gene, exon, CDS, etc.) and chromosome. This approach significantly reduces the search space when identifying nearest features, as only features on the same chromosome need to be considered. Each feature collection is further organized into separate files sorted by start and end positions, enabling efficient binary search operations when determining proximity relationships.

During decomposition, peakScout parses a GTF annotation file and extracts records for each supported feature type: gene, transcript, exon, CDS, start codon, stop codon, and UTR. For each combination of feature type and chromosome, two sorted CSV files are written: one sorted by genomic start position, and one sorted by end position. These files store feature coordinates alongside identifiers such as gene name and gene ID and serve as the reference index for all downstream nearest-feature queries.

The nearest-feature detection algorithm employs a bidirectional search strategy that simultaneously evaluates upstream and downstream features while accounting for potential overlaps. This approach is efficient as it leverages the pre-sorted nature of both the decomposed reference data and the input features, allowing for rapid identification of nearest features even for datasets containing thousands of peaks and reference features.

The nearest-feature algorithm operates in O(m log n) time per chromosome, where m is the number of query features and n is the number of reference features. Binary search is applied independently to the sorted start-position and end-position arrays, and the k nearest features are selected by merging upstream and downstream candidates. Input coordinates follow the BED convention (0-based, half-open) and are converted to 1-based closed intervals on load to match the internal coordinate system. The choice of binary search over pre-sorted arrays was made deliberately to minimize external dependencies and maximize portability. Alternative approaches, such as interval trees or tabix-indexed files, offer similar or superior asymptotic performance for large reference datasets, but require additional indexing infrastructure or compiled dependencies.

### 2.1 Supported operations

Following the decomposition step, users may proceed with one of two analytical functions: peak2gene or gene2peak. The peak2gene function identifies the nearest annotated genes from the reference dataset to a user-defined set of genomic peaks. Conversely, the gene2peak function determines the nearest peaks relative to a specified list of genes. Both functions provide options for defining peak boundaries and proximity constraints. Users can choose between native peak boundaries (as defined by the peak caller), peak summits, or artificial boundaries with user-defined extensions, as well as specify distance constraints for upstream and downstream features, ensuring that only regions deemed relevant are reported.

By default, peakScout uses peak caller native peak boundaries, preserving the original signal extent without modification. A boundary option extends a fixed distance bi-directionally from the summit, useful when uniform window sizes are required for downstream comparison. Distance constraints (upstream and downstream bounds) are disabled by default, meaning all nearest features are reported regardless of genomic distance; users may apply these constraints to restrict results to biologically plausible regulatory ranges.

When a candidate cis-regulatory element (cCRE) annotation is provided, peakScout performs an overlap step after nearest-feature detection. Results are annotated with a Boolean overlap flag, semicolon-delimited ENCODE cCRE accession identifiers, and cCRE type classifications (PLS, dELS, pELS, CTCF-only, DNase-H3K4me3). For hg38 and mm10, peakScout downloads the ENCODE cCRE Registry automatically.

peakScout supports multiple peak file formats including MACS2 (both Excel and BED formats), SEACR output and BED6, which provides wide support for most peak-calling outputs. This enables seamless integration with various peak calling workflows without requiring users to perform format conversions. A single-column gene list can be provided to determine what peaks are nearest to genes of interest. peakScout includes pre-generated reference files for commonly studied organisms: *C. elegans* (WBcel235), *Drosophila* (BDGP6.54), zebrafish (GRCz11), *S. cerevisiae* (R64-1–1), *X. tropicalis* (v10.1), mouse (mm39 and mm10), human (hg38 and hg19), and pig (Sscrofa11.1). Reference GTF files are from Ensembl (Dyer *et al.* 2025) and GENCODE (Mudge *et al.* 2025) and the decomposed references are available via a public S3 bucket.

### 2.2 Feature detection and proximity analysis

peakScout considers the full genomic context by using a bidirectional search strategy that simultaneously evaluates upstream and downstream features. Feature overlaps are handled to ensure that genes directly overlapping with peaks are prioritized in the results. For proximity constraints, a maximum distance threshold for upstream and downstream features can be set, allowing researchers to fine-tune the results based on their needs. Furthermore, peakScout further optimizes the search process by filtering reference features based on these distance constraints before performing the detailed proximity analysis. Through binary search operations, it also ensures that, after accounting for potential overlaps, searches for upstream and downstream nearest features begin at the closest possible location. These pre-filtering steps significantly reduce computational overhead.

### 2.3 Output generation and visualization

**Figure 1:**
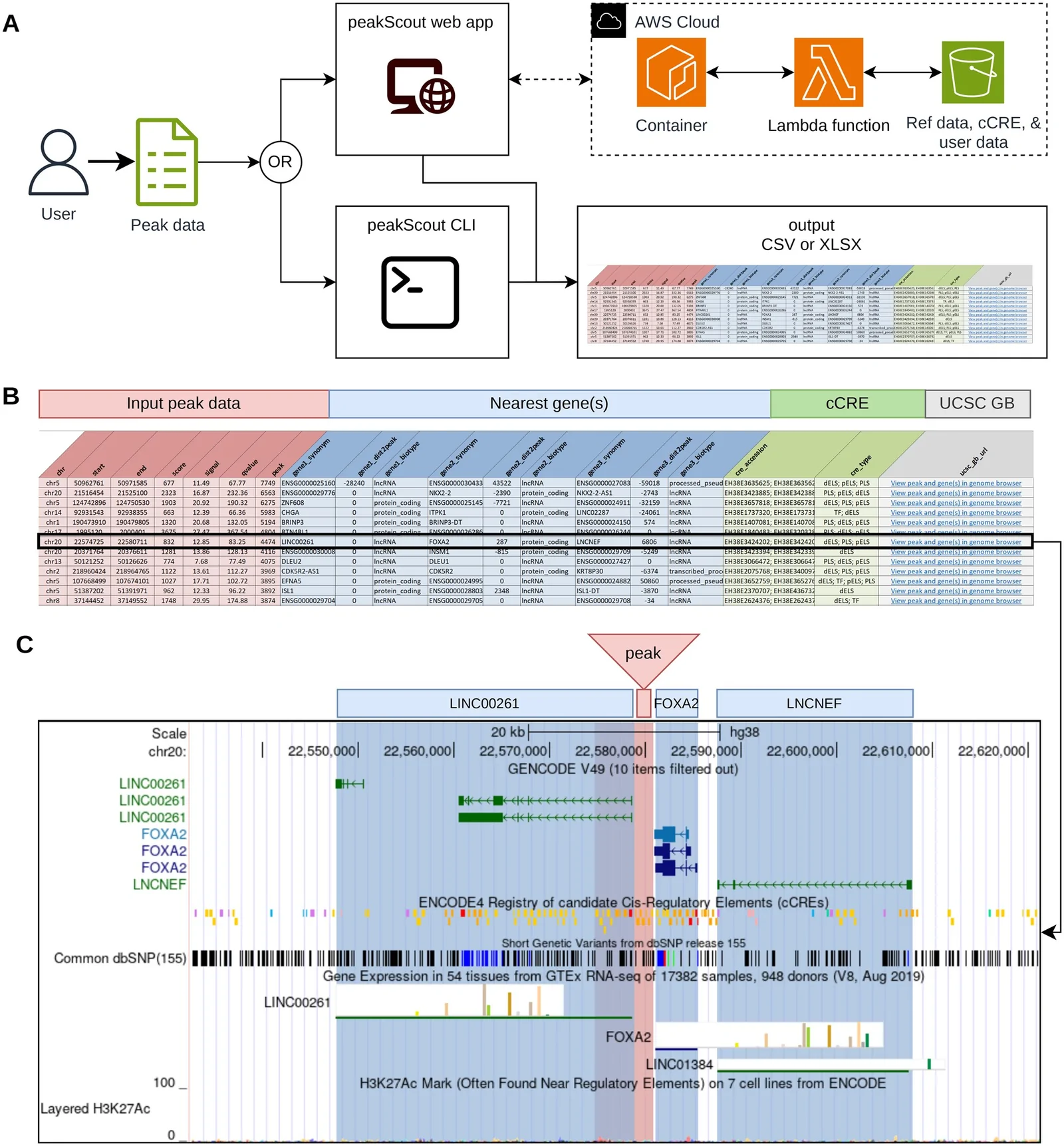
Overview of the peakScout workflow. (A) peakScout can be run either through a web application or a command-line interface (CLI); both accept the same user-supplied peak file (e.g. a BED/narrowPeak file from a ChIP-seq, CUT&RUN, or ATAC-seq experiment) and basic parameter settings. The output is a tabular result file (CSV or XLSX) annotating each peak with its nearest gene(s), regulatory context, and a genome-browser link. (B) Schematic of the output table structure. For each input peak (chr, start, end, score, signal, *q*-value, peak ID), peakScout reports the k nearest genes selected by the user (here, *k* = 3), including each gene’s identifier, distance from the peak, and biotype; the overlapping or nearby ENCODE candidate cis-regulatory element(s) (cCRE accession and type); and a hyperlink to view the peak and its neighboring genes directly in the UCSC Genome Browser. The highlighted row shows an example peak from a MAFB CUT&RUN dataset, located at chr20:22 574 725–22 580 711 (hg38). Its nearest genes are the lncRNA LINC00261 (0 bp, overlapping), FOXA2 (287 bp away, protein-coding), and the lncRNA LNCNEF (6806 bp away), and the peak coincides with cCREs annotated as distal and proximal enhancer-like signatures (dELS, pELS) as well as a promoter-like signature (PLS). (C) UCSC Genome Browser view of this region, generated via the link in (B), showing the peak’s position relative to LINC00261, FOXA2, and LNCNEF, along with ENCODE cCREs, common dbSNP variants, GTEx expression tracks, and layered H3K27Ac signal. The peak sits just upstream of FOXA2, overlapping enhancer- and promoter-like cCREs and a region of elevated H3K27Ac signal, consistent with a cis-regulatory element controlling FOXA2 transcription.

peakScout provides output in both CSV and Excel formats, with the Excel output including additional formatting for improved readability. As shown in Fig. 1, this includes comprehensive information about each peak or gene, including (i) genomic coordinates (chromosome, start, end), (ii) feature identifiers (peak name or gene name) and genomic distance for the k-nearest features, (iii) overlapping cCRE identifiers and types, and (iv) a pre-formed URL to the UCSC genome browser (for supported genomes) that links to a genomic view of the peak and nearest features, color-coded for improved interpretability. This rich output format enables researchers to more easily identify potential regulatory relationships and prioritize candidates for further experimental validation.

### 2.4 Interfaces

For use as imported functions or at the command line, peakScout can be installed as per the instructions on the GitHub website and dependencies installed using pip or uv, commonly used Python package managers.

#### 2.4.1 Use as a python library

To use peakScout functions within a typical Python script, one can import the peakScout functions (peak2gene, gene2peak, or decompose_ref) and use as desired in their program or workflow.

#### 2.4.2 Use via a website and serverless cloud computing

We have also deployed peakScout as a containerized serverless application on AWS Lambda, removing the need for users to install or maintain any software locally. The analysis environment, including all Python dependencies and reference genomes, is packaged as a Docker image and retrieved on demand from Amazon S3, eliminating the cost and maintenance burden of a persistently running server: a comparably provisioned always-on virtual server (e.g. AWS Lightsail) costs ∼$20/month regardless of usage, whereas Lambda bills only for actual invocations. As shown in Fig. 1, users interact through a lightweight web client: peak files are uploaded directly to Amazon S3 via a presigned URL, bypassing Lambda’s request payload limit entirely, and only a small job descriptor is sent to the Lambda endpoint to trigger analysis. This design supports file sizes far larger than the payload constraints of a typical serverless request cycle. Once triggered, containers execute peak2gene or gene2peak against the requested reference genome and write compressed results back to S3 for immediate preview or download via a second presigned URL. All intermediate files are discarded after execution, so each invocation is stateless and fully reproducible regardless of prior usage. A scheduled keep-warm invocation prevents the container from becoming idle, so users consistently experience the same latency reported in our benchmarks (Section 2.3) rather than a cold-start penalty. For a representative large input (a 15 MB MACS2 file containing 65 000 peaks), peakScout completes in ∼3–5 s for CSV output and 15–20 s for Excel output. We approximate the cost per query to be under a tenth of a cent, and AWS offers a generous free tier as well. The user-facing web client itself is a static HTML, CSS, and JavaScript application hosted at no cost via GitHub Pages. Since peak files are uploaded directly from the browser to Amazon S3 via a secure presigned URL and results are retrieved the same way, no user data ever transits or resides on GitHub’s infrastructure at any point in the workflow. The web version of peakScout is available at https://vandydata.github.io/peakScout/.

#### 2.4.3 Use as a command line tool

Several examples for peakScout utilization at the command line are provided in the GitHub README file. In short, one would simply type peakScout, followed by the desired function (peak2gene, gene2peak, or decompose_ref), and finally the required and optional parameters for that analysis. Example output is shown in Fig. 1C.

To evaluate runtime scalability, we benchmarked peakScout peak2gene against BEDTools closestBed (v2.31.0) using synthetic peak sets of 1000, 10 000, 50 000, and 100 000 peaks against 61 386 hg38 GENCODE v39 gene annotations. peakScout completed queries in 4.2–5.5 s, with much of the elapsed time attributable to Python interpreter startup and reference file loading rather than nearest-feature search itself. BEDTools closestBed completed equivalent operations in 0.06–0.64 s. Both tools completed within seconds for dataset sizes typical of ChIP-seq, CUT&RUN, and ATAC-seq experiments (generally 5000–30 000 peaks), so runtime is not a practical differentiator. Notably, the BEDTools timing excludes preprocessing steps required for real-world use such as MACS2/SEACR format conversion and coordinate sorting, whereas peakScout handles these steps internally as a single command.

## 3 Conclusion

While originally designed for CUT&RUN, CUT&TAG, and ChIP-seq, peakScout’s flexible input requirements and support for popular peak caller outputs (MACS2, SEACR) make it broadly applicable to other genomic methods that generate coordinate-based regions of interest, including ATAC-seq, DNase-seq, and FAIRE-seq experiments. The lack of advanced parameters is intentional to make things easy and accessible for all non-technical users, or those who desire a first pass at results before engaging in deeper bioinformatics analysis.

## Supplementary Material

btag653_Supplementary_Data

## Data Availability

We have made available preprocessed gene annotation references for common organisms, publicly available and cloud-ready at s3://cds-peakscout-public/. Example peakScout outputs are available as supplementary data 1 (XLSX) and supplementary data 2 (CSV).
