# Supplementary material for: peakScout—a biologist-friendly tool for bidirectional peak-gene mapping
Source: Bioinformatics. 2026 Sep 7;42(9):btag653. doi: 10.1093/bioinformatics/btag653 (PMC13587829; doi:10.1093/bioinformatics/btag653)
Supplement: Supplementary file 1 [file btag653_supplementary_data.zip › peakScout_Table1_supp_20260717.docx]

**Supplemental Table 1.** Comparison of peakScout with commonly used genomic peak annotation tools

| **Feature** | **peakScout** | **BEDTools** | **GREAT** | **ChIPseeker** |
| --- | --- | --- | --- | --- |
| **Peak-to-gene mapping** | Yes | Yes (closestBed) | Yes | Yes |
| **Gene-to-peak mapping** | Yes | Requires custom scripting | No | No |
| **Web interface** | Yes | No | Yes (web only) | No |
| **No installation required** | Yes (web) | No | Yes (web only) | No (R/Bioconductor) |
| **Native MACS2/SEACR input** | Yes | No (BED conversion required) | No (BED conversion required) | Partial |
| **k-nearest features** | Yes (user-defined) | No (1 closest) | No | Yes |
| **CRE overlap annotation** | Yes | No | No | No |
| **Output format** | CSV, Excel | TSV | HTML, tables | R objects, CSV |
